# Heavy Metal Ion Removal: A Global Review of Wastewater Treatment Technologies

**DOI:** 10.3390/ijms27041741

**Published:** 2026-02-11

**Authors:** Nicoleta Sorina Nemeș, Adina Negrea, Mihaela Ciopec, Petru Negrea, Narcis Duţeanu, Daniel Marius Duda-Seiman

**Affiliations:** 1 Research Institute for Renewable Energies—ICER, Politehnica University Timisoara, Gavril Musicescu Street No. 138, 300774 Timisoara, Romania; 2 Faculty of Chemical Engineering, Biotechnologies and Environmental Protection, Politehnica University Timisoara, Victories Square No. 2, 300006 Timisoara, Romania; mihaela.ciopec@upt.ro (M.C.); petru.negrea@upt.ro (P.N.); narcis.duteanu@upt.ro (N.D.); 3 Department of Cardiology, Victor Babes University of Medicine and Pharmacy Timisoara, 2 Eftimie Murgu Square No. 2, 300041 Timisoara, Romania; daniel.duda-seiman@umft.ro

**Keywords:** wastewater, metal ions toxicity, human health, removal processes

## Abstract

This review addresses the escalating global water crisis driven by water pollution, especially by heavy metal ions, a consequence of rapid industrialization and population growth. Due to their high toxicity, solubility, and persistence, heavy metals pose a severe threat to human health and ecosystems through bioaccumulation. The analysis highlights a strategic shift in wastewater management from simple elimination of the toxics metal ions to the recovery of metal ions with economic value. Given the increasing complexity of industrial effluents, the scientific community is intensifying its focus on evaluating the technical and financial feasibility of various treatment technologies. Significant research is being conducted to address these environmental issues, and innovative technologies are being developed to enhance the quality of water contaminated by metal ions. On the other hand, to prevent pollution, plans containing several barriers must be established, including management, economic, and technical ones. Ultimately, the reuse of treated wastewater is the only viable long-term solution for securing global drinking water supplies. A new analysis focused on the transition from traditional, inefficient, and costly wastewater treatment to advanced, resource recovery-oriented systems is essential. The current perspective shows a clear need to advance beyond synthetic laboratory studies to real-world applications while addressing operational barriers to support a circular economy based on simple disposal of the toxic metal ions to the recovery of metals with economic value (e.g., copper, gold, silver, rare metals). Also, although the field has been explored, a new review is imperative because current technologies that show high efficiency (up to 99%) in the removal of toxic metal ions (adsorption, membrane filtration, electrochemical processes) face major challenges, such as the formation of large volumes of toxic sludge, membrane fouling, and high operating costs.

## 1. Introduction

It is safe to say that “life depends on water.” All living things, including people, animals, and plants, depend on water, which is also a vital resource for the economy. Water is also necessary for the climate regulation cycle. However, natural ecosystems are under stress from urban sprawl, intensive agriculture, pollution, and climate change. Environmental protection and the management of water resources are top priorities for the European Union (EU). EU encouraged the development of a worldwide and cohesive approach to water law in 2000 by introducing policies and legislation in a framework document known as the “Water Framework Directive” (WFD) [1]. Through the Europe 2020 policy, the European Commission enforces certain guidelines and places a strong emphasis on the prudent use of water resources. Concerns regarding the protection of water sources revolve around managing environmental, climate, and regulatory challenges to ensure sufficient quality and quantity of water for all. Therefore, environmental policy helps to accomplish the overall goals of the plan to turn Europe’s economy into a sustainable one built on greater understanding and the effective use of the continent’s natural resources [2,3].

This review is, therefore, fundamental in the context of a global water crisis, where heavy metal pollution has become a severe and often uncontrollable problem due to relentless industrial growth and population explosion. The need for a new critical analysis is supported by the unique characteristics of heavy metals: high toxicity, particular solubility in aquatic environments and their persistence, which leads to bioaccumulation through the food chain, directly threatening human health and ecosystems. Currently, the reuse of treated wastewater is considered the only viable solution to generate additional sources of drinking water. The unlimited variety of metal ion contaminants, as well as their detrimental impact, are the fundamental features of industrial wastewater. They are included in the category of “toxins” in the environment. The issue of heavy metal toxicity is turning into a public health concern on a global scale, impacting not only the population but also the development of purification methods, preventing exposure to them, and, in cases where intoxication has already occurred, the methods for diagnosis and treatment.

**Figure 1:**
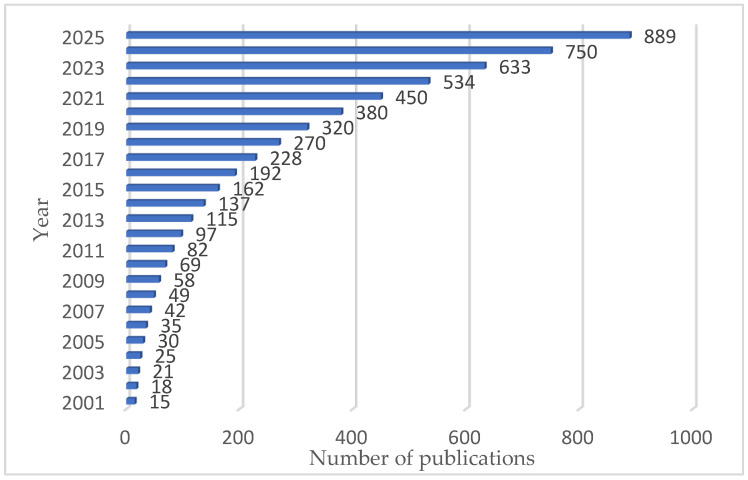
Number of publications related to wastewater treatment technologies (Scopus/Web of Science database).

The increased interest in the use of various technologies in wastewater treatment is justified by the exponential increase in the number of published papers in the field of ion removal on wastewater treatment, according to the Scopus/Web of Science database (Figure 1).

The increasing number of scientific articles in this field demonstrates interest in this subject and the recognition of the importance of knowing in detail the advantages and disadvantages of various water treatment processes in addressing complex wastewater treatment or purification problems.

Therefore, depending on the nature and specificity of wastewater (natural or anthropogenic ones), its treatment may involve either methods of recovering pollutants in various forms (ionic or metallic) that have economic value or methods of elimination. From a technical and financial perspective, recovering metal ions from wastewater can be successful for a variety of metal ions, including platinum group metals (PGMs) (ruthenium (Ru), rhodium (Rh), palladium (Pd), osmium (Os), iridium (Ir), and platinum (Pt)) and precious metals (gold (Au) or silver (Ag)) or some ions results from industrial activity, especially copper mining activities (Cu). When it comes from acid mine drainage (AMD) and tailings discharge, or the recovery techniques are not advised, contaminants must be eliminated using procedures that agree to environmental protection regulations. Because these ions are non-biodegradable and have a tendency to collect in nature beyond the limits permitted by law, certain metal ions, such as heavy metals, are known to be carcinogenic agents and can pose a major hazard to humans and related organisms [4,5,6,7]. Due to its intrinsic toxicity, tendency to build up in the food chain, and, particularly, its slow rate of disintegration, it stands for metals that can damage the ecosystem even at low quantities [8]. The main metal ions that can contaminate large areas of surface water are those of heavy metals (as bismuth (Bi), copper (Cu), zinc (Zn), cadmium (Cd), mercury (Hg), lead (Pb), chromium (Cr), iron (Fe), nickel (Ni), molybdenum (Mo), cobalt (Co), manganese (Mn), vanadium (V), gallium (Ga)). They are present in sludges, fertilizers, pesticides, municipal waste, and mining residues [9,10,11,12,13,14,15].

**Figure 2:**
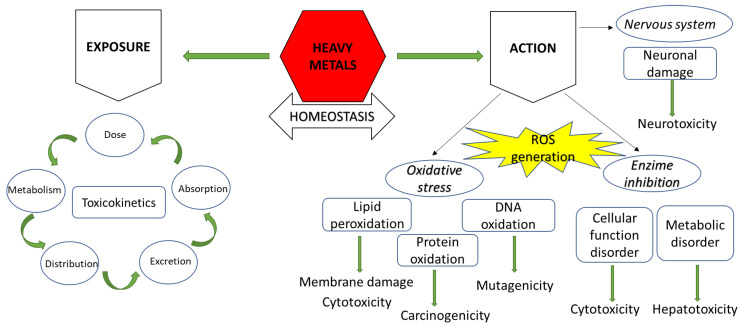
Heavy metals toxicity.

So many studies have concentrated on these toxicity studies since drinking water that are frequently contaminated with heavy metals are consumed [16,17,18,19,20]. Reactive oxygen species (ROS) production, together with the metals’ enhanced capacity to inhibit enzyme activity and disrupt antioxidant defense mechanisms, is a crucial mechanism by which the harmful effects of heavy metals on the human body are realized [21]. Because it destroys proteins, DNA, lipids, and lipid membrane structures, oxidative stress may be the primary mechanism causing carcinogenesis (Figure 2) [22,23].

Previously published papers in the field of removal of heavy metal ions from wastewater [24,25,26] focused on the mechanisms of ion removal. In the present papers, the authors examine in detail the practical drawbacks and limitations of implementing each method on an industrial scale. The literature review provides insights into the treatment processes, providing a guide to solutions that are not only theoretical but also easy to implement and sustainable. While Hussein et al. focused on nanotechnology, and Joshi Amey Anant et al. and Qasem, N.A.A. based on a traditional description of methods for removing pollutants from wastewater (adsorption, membrane filtration, chemical, electrical, and photocatalytic processes) and analyzing in detail their limitations (toxic sludge, membrane fouling), the present work correlates technical efficiency (which can reach 99%) with economic feasibility and long-term environmental impact. A significant novelty of the work is that it emphasizes the importance of recovering metals that have economic value from wastewater, transforming water treatment from a waste disposal process into one of resource recovery.

On the other hand, it is commonly known that certain heavy metals, in relatively little amounts, support healthy and functional human systems; nonetheless, the limit for activating harmful processes is quite low. Environmental, genetic, excessive-deliberate or accidental and congenital or acquired deficiencies are some of the circumstances that trigger the toxic effect of metal ions [27]. The main heavy metals, with their uses, toxic effects, and sources, are shown in Table 1.

Heavy metal toxicity makes its removal from water a serious concern, requiring the development of physical, chemical, and biological techniques to remove them from water in a selective manner [51,52]. Critical analysis of the sources reveals that, although the mechanisms of heavy metal removal are well understood theoretically, their practical application faces significant technical and economic barriers. It emphasizes that there is a major gap between laboratory efficiency (often over 99%) and industrial sustainability.

Although the field has been explored, a new review is imperative because:

(i) There is a clear knowledge gap regarding the performance of treatment methods on real wastewaters, with most current studies relying on synthetic solutions with few contaminants.
(ii) Current technologies (adsorption, membrane filtration, electrochemical processes) face major challenges, such as the formation of large volumes of toxic sludge, membrane fouling, and high operating costs.
(iii) Research needs to progress from simple disposal to the recovery of economically valuable metals (e.g., copper, gold, silver, rare metals), supporting the circular economy.

## 2. Physical, Chemical, and Biological Methods for Metal Ion Removal from Wastewater

The insoluble content of wastewater could be composed of inert substances such as rainwater drainage, dust, or sand (in the form of ballast from raw materials such as lime). However, it could also be composed of hazardous materials such as heavy metals and their compounds, which appear from the precipitation process of previous treatment operations or production processes that use catalysts. The removal of metallic ions from wastewater using physicochemical techniques is most suitable for contaminants like fine or coarse metal particles [53,54,55]. However, insoluble pollutants do not always have to be solid particles; they can also be liquids that are immiscible with water, such as oil, lubricants, colloids, and compounds with an oily consistency [56] that need to be separated before the decontamination stage. Physical separation consists of gravity concentration, flotation, mechanical screening, hydrodynamic classification, magnetic separation, and electrostatic separation, but also washing technics [53,57]. Many variables of the wastewater, including the size of the particles that are dispersed, heterogeneity, density of metal pollutants, magnetic properties, and hydrophobic qualities of the solid particle surface, affect how well physical separation works [58,59]. Physicochemical processes have many advantages over other processes, such as simple and controlled operation, flexibility, and controlled temperature. However, these processes have some disadvantages, such as high energy requirements and high costs.

**Figure 3:**
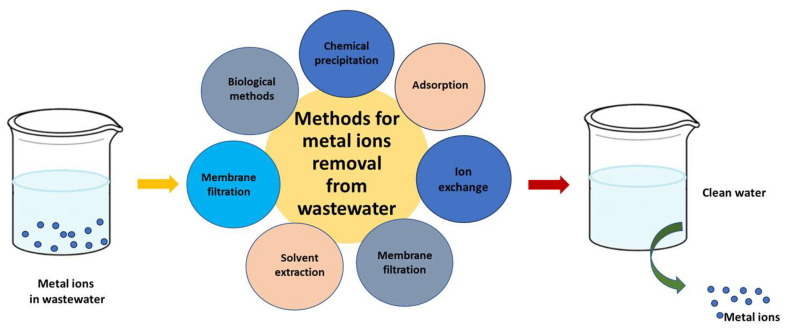
Technological methods used in metal ion removal from industrial wastewater.

Figure 3 provides a suggested framework for the most popular and widely applied techniques for removing metallic ions from wastewater.

### 2.1. Chemical Precipitation and Coagulation-Flocculation

**Figure 4:**
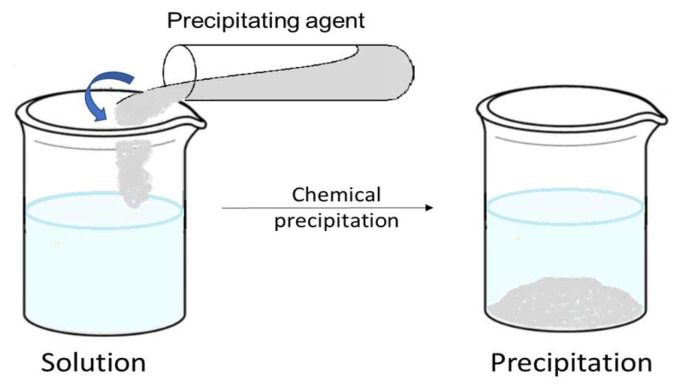
Chemical precipitation process scheme.

A schematic diagram for chemical precipitation is presented in Figure 4.

Lime, Ca(OH)_2_, caustic soda, NaOH, and other inorganic precipitation agents are the most commonly employed for precipitating metal ions as hydroxides [60]. Soda ash (Na_2_CO_3_) and sodium bicarbonate (NaHCO_3_) are typically used for carbonate precipitation, whereas sodium sulfide (Na_2_S), and sodium hydrosulfide (NaHS) are most frequently employed for sulfide precipitation. In general terms, the desired degree of disinfection determines which precipitating agent is used. It should not be forgotten that while selecting the precipitating agent, which regulates the solubility of metals in the removal process, the pH of the working solution must be taken into account [60,61].

The general mechanism of the precipitation process in the form of hydroxides is extremely simple [60,62,63]:

M^n+^ + n(OH)^−^ → M(OH)_n_ ↓ (1)

where: M^n+^—metal cation

M(OH)_n_—insoluble metal hydroxides

Generally, Cu^2+^, Zn^2+^, Ni^2+^, Pb^2+^, or Cr^3+^ is removed by the precipitation method in the form of hydroxides [26,62]. Precipitation as hydroxides has many limits despite its widespread use. First, it is well known that significant amounts of comparatively low-density sludge are produced, which complicates the disposal procedure. Second, some metal hydroxides are amphoteric, which makes it difficult to remove some metals from the combination because the pH at which one metal precipitates may also be the pH at which another metal in the mixture returns to solution. Third, the presence of complexing agents in the water prevents the metal hydroxide from precipitating, leading to inadequate processing and large quantities of potentially hazardous sludges [10,26,64,65,66]. A different kind of precipitation occurs when different sulfides are employed as precipitation agents. This results in precipitates in the form of metal sulfides, which are not amphoteric and have a significantly lower solubility than hydroxides [10,64,67]. In general, Cu^2+^, Cd^2+^, and Pb^2+^ ions can be eliminated also by precipitation in the form of metal sulfides.

The mechanism of precipitation in the form of metal sulfides is also very simple:

(2) $$2M^{n+}+nS^{2-}\to M_{2}S_{n}↓$$

where: M^n+^—metal cation

$M_{2}S_{n}$—insoluble metal sulfide.

But, because metal ions are frequently found in acidic environments and because precipitation in the form of metal sulfides can increase the amount of hazardous hydrogen sulfide vapors in acidic environments, metal sulfide precipitation also poses ecological problems [68]. As a result, neutral or basic conditions are typically used for this precipitation procedure. Furthermore, colloidal precipitates produced by metal sulfide precipitation frequently have separation issues during filtration or sedimentation procedures [10,69].

Furthermore, precipitation is a chemical process that creates particles that can subsequently be separated by another procedure like sedimentation, air flotation, filtration, and, if required, microfiltration (MF) or ultrafiltration (UF). Thus, the precipitation process can be combined with other processes like flocculation or coagulation to remove metal ions more effectively, leading to a higher level of pollutant removal. Using a chemical coagulant, the coagulation treatment destabilizes the colloidal particles and causes sedimentation. Unstable particles typically flocculate into large flocs that are subsequently deposited in the sedimentation tank after coagulation. Typical metallic ions that can be removed by this method include Cu^2+^, Pb^2+^, Ni^2+^, As^5+^, Fe^3+^, Cr^3+^, and Zn^2+^ [26,62,70]. One of the most crucial methods for treating wastewater that contains hydrophobic colloids and suspended solids is precipitation–coagulation [10].

Table 2 shows the performances regarding the removal of metal ions by precipitation or coagulation–flocculation.

As it was indicated, even if both chemical precipitation and coagulation flocculation methods exhibit extremely high removal rates for most heavy metal ions, there are some limitations in the technologies of removal. Thus, the high efficiencies reported in Table 2 (over 95–99% for most heavy metals) are based on well-established chemical principles but can be applied only in certain technical conditions, such as pH, initial pollutant concentration (metal ion), dose, and nature of the precipitation or flocculation agent.

New strategies have been investigated to create more effective and cost-effective ways for the removal of metal ions because precipitation removal procedures have a number of limitations [64,74,75,76].

**Figure 5:**
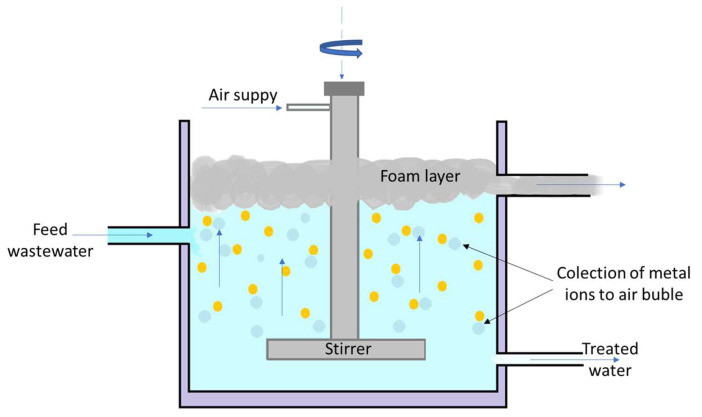
Flotation process scheme.

By adhering to certain air bubbles, solid or liquid particles or granules are extracted from wastewater through flotation. With the aid of foam, floating particles are gathered on the water’s surface (Figure 5) [73,77,78,79]. To help with the flotation process, flocculant additives such activated silica, ferric, or aluminum salts, and different organic polymers, are frequently utilized. They are used to create a surface or structure that can absorb or hold air bubbles, in addition to coagulation and flocculation processes [80].

Different flotation techniques can be identified based on how air is added: (i) Vacuum flotation, which allows bubbles to develop by dissolving air at atmospheric pressure and then allowing a pressure decrease; (ii) A water treatment method called induced air flotation (IAF) removes suspended materials like sediments or oil from wastewaters (or other waterways) to make them clearer; Electrical flotation (also known as electroflotation), vacuum air flotation, dissolved air flotation (DAF), or biological flotation [73,81,82,83]. In Table 3, performance in flotation efficiency in the process of removing metallic species is shown.

Although the metal ion removal results presented in Table 3 confirm a high selective efficiency, the applicability of these results must be viewed in the context of the transition from controlled (laboratory) conditions to industrial scale applications. The efficiency of the removal method by flotation can be supported by ion exchange and complexation mechanisms, which occur with the addition of flocculants to the water to be treated [79,90]. However, the transposition of these performances to industrial scale must consider several important limitations. First, the matrix effect (the presence of competing ions) occurs because in real effluents, the presence of other ions or competing organic matter can occupy the active sites of the flocculant, reducing the selectivity for heavy metals. On the other hand, the mechanical stability of the formed flocs is conditioned by maintaining an optimal pH [92,93], specific to each metal-flocculating agent couple, in order to obtain maximum flocculation performance.

*Critical viewpoint for chemical precipitation and coagulation–flocculation methods:*

- Although they are inexpensive methods for large volumes of water, the transformation of pollution from liquid to solid (sludge) remains the greatest environmental challenge of these technologies, which is a critical drawback. This sludge requires special treatment and storage, being itself a hazardous waste.
- The large volume of toxic sludge generated turns a water pollution problem into a hazardous waste management problem. The costs of storage in compliant landfills can quickly negate the initial savings of the treatment process.
- Certain metals become soluble again if the pH is not maintained within an extremely strict range, which makes the process unstable in fluctuating industrial streams.
- These methods (especially chemical precipitation and coagulation) become ineffective when heavy metal ions are in low residual concentrations (traces), often unable to reach the very strict limits imposed by environmental discharge regulations.

### 2.2. Adsorption Used for Metal Ions Removal from Wastewater

**Figure 6:**
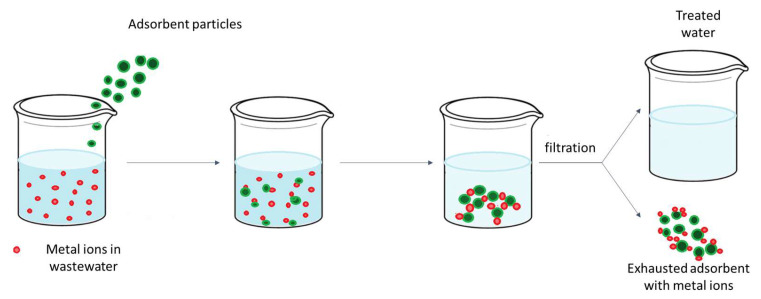
Adsorption process scheme.

To discover new materials with adsorbent qualities targeted at the removal or recovery of a specific metal ion, the research of several collectives has mostly concentrated on the process of removing or recovering metal ions from aqueous solutions by adsorption. Adsorption is the process by which a gas or a dissolved substance accumulates on the surface of a solid or liquid (adsorbent), forming a film on the surface (adsorbate) (Figure 6). Due to its affordability and ease of use, the adsorption method of removing metal ions is ideal for treating wastewater.

Adsorption is an efficient process for both advanced wastewater treatment and drinking water treatment from natural sources. It is an effective process with the benefit of selectivity toward specific components to eliminate contaminants from water in low concentrations (ppm or ppb) that are left over after other traditional purification methods have been applied. Adsorption is advised not only for the retention of heavy metal species but also for the removal of anions, detergents, nonbiodegradable chemicals (dyes, insecticides), and other hazardous materials that are challenging to retain using traditional techniques.

Adsorption is the process by which soluble substances (dissolved substances) are transferred from wastewater onto the surface of an adsorbent, which is an extremely porous material, in the form of particles. Each species can only be eliminated to a certain extent by the absorbent. When this capacity is exhausted, the adsorbent is “consumed” and must be replaced with new material [94]. Ionic contaminants in a series of effluents can be reduced, potentially eliminated, by adsorption utilizing materials with organic or inorganic adsorbent capabilities. This process can even recover or revalue important components, particularly metallic ions. In general, adsorbent materials, to be attractive, should be cheap and available [6,95,96] and, above all, to be regenerated easily, providing quantitative recovery. Because of the results, selective adsorption employing biomaterials, mineral oxides, activated carbon, waste and industrial by-products, or polymeric resins has led to a revolution in research [97,98,99,100]. Adsorption can occur in different ways, involves some different mechanisms that are summarized below.

#### 2.2.1. Adsorption Mechanisms

Based on the nature of the forces that appear during an adsorption process and involve the adsorption mechanism there are: (i) physical adsorption (physisorption), in which weak, often reversible forces appear, and (ii) chemical adsorption (chemisorption), in which strong, often irreversible bonds appear. Physical adsorption is the method by which heavy metal ions diffuse and deposit themselves in the adsorbent material’s pores without creating chemical bonds. The process depends especially on the pore size distribution and surface area of the adsorbent. In this way, expanding the pore size can increase the material’s surface area and make it easier for metal ions to diffuse, that can accelerate the kinetics of the adsorption process [101]. Chemisorption needs significant energy, being a surface process where adsorbate form strong chemical bonds (covalent, ionic, metallic) with adsorbent through electron sharing or transfer, creating a new chemical species.

**Figure 7:**
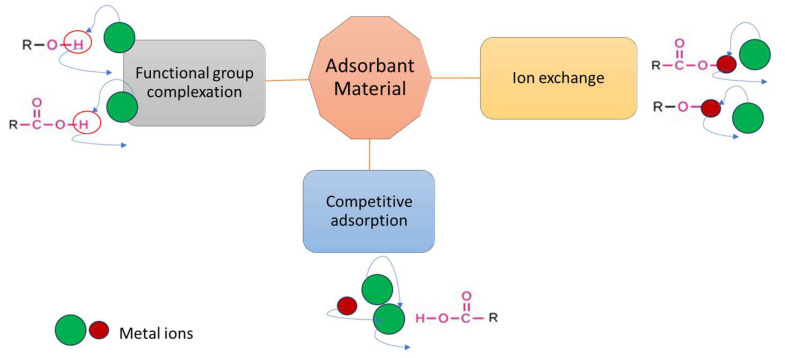
Adsorption mechanism.

Based on the specific interactions of the adsorption mechanisms (Figure 7), the functional group complexation involves functional groups (such as -OH, -COOH, -O-, and -CO-NH-) that react with metal ions (especially M^2+^) or complexes on the adsorbent surface present in aqueous solutions [102,103,104]. Metal ions form highly stable, polydentate coordination structures through chelation primarily due to the chelate effect, which is a thermodynamic phenomenon where multidentate ligands form complexes with significantly higher stability constants than similar complexes with monodentate ligands [105]. In wastewater treatment, this allows for the effective sequestration and removal of heavy metal ions (such as Cu^2+^, Ni^2+^, Zn^2+^, and Pb^2+^) by forming stable, often insoluble, complexes that are more stable than metal hydroxides or sulfites [106,107].

Also, ion exchange, especially the exchange between metal ions and protons from functional groups that include oxygen (hydroxyl or carboxyl groups) is a complex type of adsorption process. The size of the metal ion and the chemistry of the adsorbent’s surface functional group have a significant impact on the efficiency of the ion exchange process. When ion exchange is the primary adsorption process, cation exchange capacity is a crucial measure of metal ion adsorption [103,108].

From the point of view of the factors that influence the adsorption process or from equilibrium phenomena that may occur during the adsorption process in the case of multicomponent systems, competitive adsorption processes encounter. The mechanism of competitive adsorption of metals is based on the batch equilibration adsorption and adsorption kinetic studies and is influenced especially by selective affinity. In complex, multi-component wastewater treatment, such as industrial effluents, mining, or textile processing, the multi-site, multi-contaminant environment significantly impacts adsorption performance. Wastewater treatment involves competitive adsorption, where multiple ions compete for limited active sites, resulting in antagonistic or, rarely, synergistic interactions [100,109].

#### 2.2.2. Metal Ion Removal from Wastewater by Adsorption Used Carbon-Based Adsorbents

The mechanism that underlies the process of removing metal ions from water by adsorption depends mainly on the nature of the material.

Carbon-based materials (biochar, activated carbon, and charcoal) are excellent absorbents for removing metal ions from water, the processes being based on physical adsorption, ion exchange, and chemical complexation (Table 4). The difference between these materials is made by the production method, pore structure, and adsorption capacity. Of these, activated carbon, in both powders activated carbon (PAC) or granular activated carbon (GAC) forms, is recognized for its highest adsorption capacity and active surface area [110,111,112,113]. Adsorption using GAC is applied especially for the removal of organic contaminants, mainly those with toxic properties, such as dyes with and without odor, and for the removal of inorganic contaminants, such as nitrogen compounds and sulfides, as well as heavy metal species. Granular media filters, for example, sand filters, are typically used upstream of the GAC adsorbent to remove suspended solids. The use of PAC in adsorption processes occurs in the case of wastewater loaded with organic substances, or refractory, toxic, or dangerous substances that have arrived from the sedimentation container or from other containers [114]. PAC can also be added to the aeration basin of a system where activated sludge is present, which is used in microbiological processes to increase the efficiency of adsorption processes. PAC is not normally regenerated and becomes part of the sludge to be deposited, increasing the amount of solid waste, which presents a disadvantage [115].

The absorption of metal ions from aqueous solutions on activated carbon is more complex than the absorption of organic compounds because ion exchange affects the removal kinetics from the solution. The adsorption capacity depends on the properties of the activated carbon, namely: chemical properties, temperature, pH, ionic strength, etc. There are many commercially available forms of activated carbon, but few are selective for metal ions [118,119]. Although activated carbon is an adsorbent with a very well-developed microporous structure that determines a huge specific surface area that allows the efficient removal of many heavy metal ions, its process of obtaining, by pyrolysis followed by activation, is expensive and requires a lot of energy for production [120]. Therefore, biochar seems to be considered a sustainable solution [121,122] because it can be obtained from agricultural waste, manure, and vegetable residues; that is, materials with no other value, and its obtaining is achieved with lower production costs. Charcoal has a lower adsorption capacity due to its reduced porosity resulting from its dense structure.

#### 2.2.3. Metal Ion Removal from Wastewater by Adsorption Using Materials with Inorganic or Polymeric Support Structures

As was already mentioned, the selection of adsorbent material is usually a complex decision. The adsorptive capacity of absorbent materials that are effective in the laboratory may fail on an industrial scale. Thus, the choice of adsorbent material in correlation with water treatment technology is very important. Although a high adsorption capacity is necessary, without superior selectivity, adsorption cannot be applied on an industrial scale. Therefore, increased research has aimed at obtaining new adsorptive materials that possess both properties. However, to have practical value, selectivity remains the essential characteristic. For the selectivity of the adsorption process, all types of solid, inert supports can be modified physicochemically by various functionalization methods with different extractants that have active groups (S, N and P) to improve the adsorbent properties and, thus, to have a higher degree of removal of metal ions. Various types of functionalizing extractants like ionic liquids, amino acids, organophosphoric extractants, and organic compounds with chelating extractant functional groups, such as crown ethers, have been used to obtain an increased efficiency of the adsorption process [123,124,125,126]. Crown ethers have considerable potential to be used as extractants with increased selectivity for metals due to their ability to form stable complexes with their ions, but also due to the fact that metal ions penetrate into the circular cavity of the crown ether forming rings with a three-dimensional structure [127,128]. For the use of crown ethers as extractants, a small amount of crown ether is needed, which indicates the economic efficiency of the obtained material, even if it is known that crown ethers are relatively expensive reagents. The efficiency of such a system leads to the development of new materials with solid-state use, capable of high selectivity toward certain metal species [127,129,130]. To apply these methods, the extractant must be liquid or be kept in a liquid state by the addition of solvent. The extractant and the solvent, in which the extractant is dissolved, must have minimum solubility, the support must be prepared for impregnation, and the functionalization method must not change the properties of the extractant or of the support [131,132].

Throughout the research, special attention was paid to inorganic solid supports (i.e., SiO_2_, MgSiO_3_, Al_2_O_3_) that were functionalized with different extractants that allowed the recovery of some species like Cs^+^, Cu^2+^, Pb^2+^, La^3+^, Pt^4+^, or Pd^2+^ (Table 5).

Also, porous polymeric supports such as aromatic copolymers styrene-divinylbenzene—(XAD-2, XAD-4, XAD-12, XAD-16), aliphatic ester-type copolymers (XAD-7 etc.) or acrylic ester-type copolymers (XAD-8) [130,134,137,140,141,142,143,144,145,146]—can be functionalized with organophosphorus acid (DEHPA) or other phosphonic/phosphinic groups for the selective separation of rare earth elements, such as Nd^3+^, La^3+^, and Eu^3+^, platinum group elements, such as Au^3+^, Pt^4+^, Pd^2+^, and Ru^4+^, or other elements, such As^3+^, As^5+^, Zn^2+^, Cd^2+^, Cs^+^, Pb^2+^, and Cu^2+^.

Table 6 highlights the adsorption capacity of materials with polymeric support structures.

The use of porous polymeric supports, with a large specific surface and good mechanical stability, is much more suitable for the removal of toxic elements from dilute solutions due to the high kinetic speed, ease of regeneration, and high adsorption capacity [157,158].

Polymeric and inorganic adsorbents represent sustainable, versatile, and often complementary materials in water remediation, each offering distinct advantages based on their structural, chemical, and economic profile. While they share some overlapping characteristics, they differ in their origin, structure, and specific adsorption mechanisms. Polymeric adsorbents are optimal for low-cost, sustainable applications, particularly in treating agricultural wastewater or heavy metal removal, provided they are sufficiently modified for stability. Inorganic adsorbents are preferred for high-performance, high-capacity, and high-selectivity applications in industrial water treatment thanks to their superior strength and tailorable chemistry.

Current research heavily favors hybrid composites (Table 7). Combining organic frameworks (e.g., biochar, MOFs) with polymeric matrices (alginate, chitosan) is becoming the standard for achieving both the high capacity of organic materials and the structural durability and processability of polymer matrices. For example, modern nanobiochar composites now use polymer coatings to prevent particle leaching while maintaining high pollutants removal rates [159,160].

To improve recycling capacity, mechanical strength, and the ability to function effectively in complex aquatic environments, these hybrid materials have proven to be promising because their structure allows the individual properties of each matrix to be combined, resulting in material with properties directed towards the intended purpose. Modern hybrid composites utilize polymer coatings to encapsulate the particles, preventing them from leaching into water systems while maintaining high efficiency in removing pollutants, such as heavy metals, dyes, and pharmaceuticals.

*Critical viewpoint for adsorption method:*

- Adsorbent regeneration is difficult to realize; desorption agents can destroy functional groups, reducing adsorption capacity in subsequent cycles.
- Although nanoadsorbents have a huge specific surface area, they tend to agglomerate quickly due to Van der Waals forces, losing their reactivity.
- The sensitivity of the method to environmental conditions (pH and temperature) is a major drawback because at too low pH, hydrogen ions occupy the active sites, repelling heavy metals. This requires constant monitoring and adjustment of the pH, which adds technological complexity.
- For some materials, the process of reaching equilibrium can take hours.
- In real wastewater, the presence of several ions leads to competition for active sites, with the mechanism being often disturbed by background ions.

### 2.3. Membrane Filtration Used for Metal Ions Removal from Wastewater

**Figure 8:**
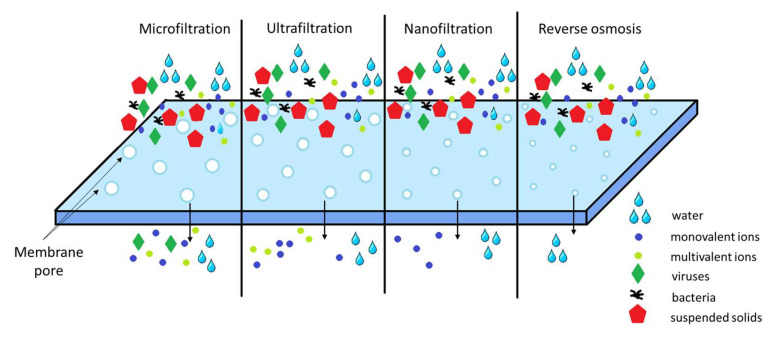
Membrane filtration process scheme.

Membrane filtration has received considerable attention for advanced inorganic effluent treatment because it can remove not only suspended solids and organic compounds, but also inorganic contaminants such as metal ions as heavy metal species. Membrane filtration techniques effectively remove various heavy metal ions from water and wastewater, including copper, nickel, lead, cadmium, zinc, silver, cobalt, and cadmium ions. Depending on the particle size that can be retained, different types of membrane filtration system can be used to remove metal ions from the solution, such as microfiltration (MF), ultrafiltration (UF), and nanofiltration (NF), but also reverse osmosis (RO) (Figure 8) [166,167,168].

The membrane filtration process is usually applied with the help of a tangential flow, i.e., the permeate flow is directed perpendicularly to the feed flow. Impurities remain in the feed stream, which, reducing in volume, leaves the membrane system in the form of a concentrated residue stream. Nanofiltration (NF) has unique properties between the ultrafiltration (UF) and reverse osmosis (RO) membranes. Its separation mechanism involves steric effects (sieving) and electrical effects (Donnan) [169,170].

MF, UF, and NF are technological processes based on the use of the membrane that segregates a liquid, which passes through a membrane, into the permeate part (which passes the membrane) and the concentrate part (retained by the membrane). The driving force of the process is the difference in pressure across the membrane. The membranes used for MF and UF are “pore-type” membranes that function as a filter. The liquid part and particles of molecular size can pass through the pores, while suspended particles, colloidal particles, bacteria, viruses, and even large macromolecules are retained. NF removes most organic molecules, a good part of viruses, most natural organic matter, and several salts. The characteristic of NF is that it uses a membrane with a higher permeability of 0.001–0.05 µm. The required water inlet pressure must be greater than 0.7 bar [171,172].

RO separation can provide some complex functions and processes, in addition to strict separation, which are recommended for high-tech uses. However, techniques based on RO are generally expensive [173,174,175]. Factors that play a key role in the use of RO membranes include pretreatment with UF or MF to prevent blocking of the membrane pores. RO is a filtration method used to remove up to 99% of organic and inorganic impurities as small as 0.0001 µm. This filtering method is currently the most effective. Water filtered by RO has a more pleasant taste and a reduced level of minerals in the water, being closer to the composition of distilled water. Another advantage of RO water purification is that, as a result, all species of heavy metals, nitrates, pesticides, or herbicides, which may be in tap water, will be removed. For example, in the case of arsenic removal, since it represents an advanced removal technique, both RO and electrodialysis have a rather low efficiency if As(III) is not oxidized to As(V) beforehand, which presents a much higher efficiency in removing As(V) than As(III) [176,177,178].

Membrane separation materials for heavy metal removal have moved beyond simple size exclusion to highly tailored, multifunctional systems, with current research focusing on Thin-Film Composites (TFC) or Mixed Matrix Membranes (MMMs) to achieve high flux, selectivity, and fouling resistance. While reverse osmosis offers 98–99.9% efficiency for heavy metals, its high energy demand has shifted research toward NF for sustainable, cost-effective treatment.

In membrane technology, especially for RO and NF, membranes with high permeability (high flux) tend to have low selectivity (low retention) and vice versa. This is because, with increasing flux, a larger number of contaminants are allowed to pass, which results in reduced selectivity. Because flux is inversely proportional to the thickness of the selective layer, making the layer as thin as possible allows for high flow even if the material itself is very dense and selective. To improve membrane filtration performance, recent research shows the incorporation of porous materials (metal organic frameworks, zeolites, carbon nanotubes) into the polymeric matrix (polyvinylidene fluoride (PVDF), polyethersulphone (PES)) [179,180], which allow increased solute flux without affecting selectivity. Modified membranes enhance flux, also increase hydrophilicity and provide high-specific-surface-area adsorption sites, while adsorptive membranes combine filtration with functional groups (e.g., amine, carboxyl, thiol) that chemically bind to heavy metals, overcoming the slow kinetics of traditional adsorption. Chemical modification by grafting is also being studied to induce specific affinities, allowing a solubility that compensates for diffusivity limitations [100,181,182].

However, NF is considered a promising technology, using electrostatic repulsion (Donnan effect) to reject divalent heavy metal ions (Pb^2+^, Cd^2+^, Cu^2+^) with 80–99% efficiency while allowing water to pass.

Recent studies compare membrane filtration removal capacities for Cu^2+^, Pb^2+^, and As^3+^ or As^5+^, emphasizing material innovations like TFC, nanoparticles, and carbon nanotubes that boost selectivity (Table 8).

*Critical viewpoint for membrane filtration methods:*

- Performance degradation: Fouling, caused by the accumulation of metal ions, microorganisms, and organic matter on the membrane surface, leads to a significant decrease in permeate flux and an increase in operating pressure, requiring frequent chemical cleaning, reducing efficiency over time, and shortening the life of the equipment.
- Require rigorous water pretreatment to prevent damage to the filter surfaces.
- Generate a toxic concentrate that requires subsequent, specialized management.
- Although operating costs may be lower than some traditional methods, the high initial investment for membranes and the high operational costs for cleaning and replacement are significant barriers.
- Material limitations: Polymer membranes, which are flexible and cost-effective, are vulnerable to chemical degradation and fouling, while robust ceramic membranes are more expensive.

### 2.4. Ion Exchange Used for Metal Ion Removal from Wastewater

**Figure 9:**
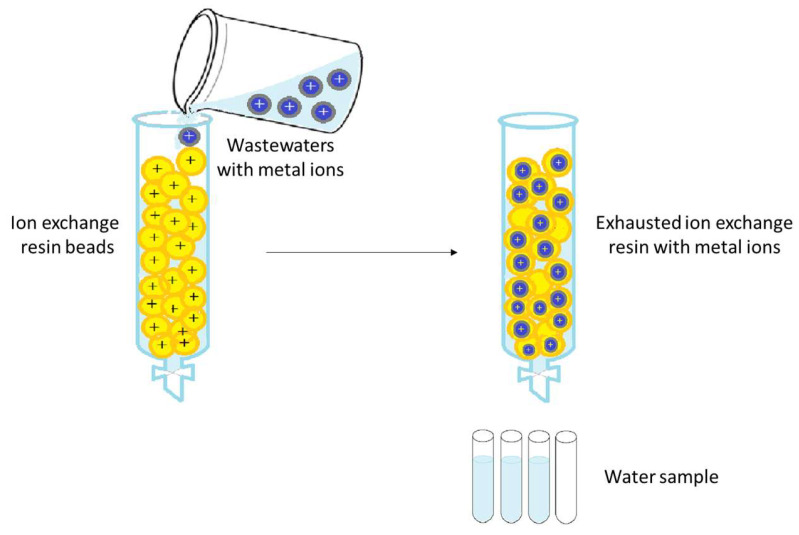
The ion exchanger process scheme.

In addition to membrane filtration, ion exchange is also one of the most widely applied treatments worldwide for wastewater loaded with various metal ions [189]. Ion exchange means removing hazardous ionic constituents from wastewater and replacing them with “more acceptable ions” from an ion-exchange resin, where they will be temporarily retained and then released into a liquid used for regeneration or backwashing (Figure 9).

For ion exchange, macroporous granular resins with anionic or cationic functional groups are usually used, such as: (i) gel strong acid cation (SAC) ion exchange resin, which neutralizes strong bases and transforms neutral salts into their corresponding acids; (ii) gel weak acid cation (WAC) ion exchange resin, which is capable of neutralizing strong bases and is used for desalkanization; (iii) Strong base anion (SBA) exchange resins, which neutralize strong acids and transform neutral salts into their corresponding bases; (iv) Weak base anion (WBA) exchange resin, which neutralizes strong acids and is used for partial demineralization [190].

In Table 9, the performance of ion exchange resin efficiency in the process of removing metallic species is shown.

*Critical viewpoint for Ion exchange used for metal ions removal:*

- Ion exchange resins are highly susceptible to fouling by suspended solids, iron, manganese, organic matter, and oils. This requires extensive and expensive pretreatment to prevent blocking of the active exchange sites.
- Although effective at high concentrations, the efficiency of the resins decreases in complex industrial wastewater containing high levels of non-toxic ions (e.g., Ca^2+^, Na^+^), which compete with the metals to be removed for the active sites.
- The resins become saturated and then must be regenerated using chemicals, thus producing a small volume of highly concentrated hazardous liquid waste.
- Requires high operating costs for industrial-scale processes (specialized resins, regular consumption of regeneration chemicals, resin replacement costs).
- If the resin is not regenerated properly, the metal-loaded resin or regeneration waste can lead to secondary environmental pollution.

### 2.5. Electrochemical Treatment Techniques Used for Metal Ions Removal from Wastewater

**Figure 10:**
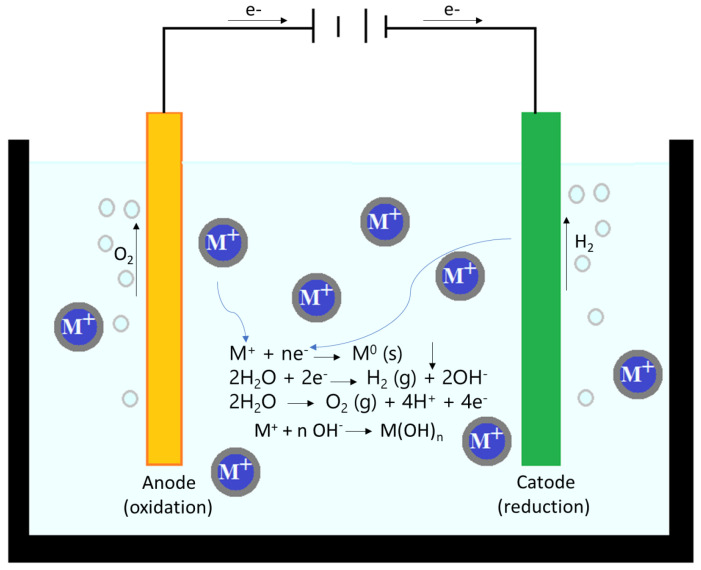
Electrochemical process scheme.

Electrochemical technologies exhibit high efficiency in treating wastewater contaminated with metal ions. These technologies offer several benefits, including profitability, operational flexibility, and even environmental friendliness [198,199,200]. Electrochemical treatment of wastewater containing metal ions is carried out mainly by electrocoagulation with soluble anodes (Figure 10) [198,201]. However, the deposition of metals from wastewater by electrochemical processes is in increasing use nowadays due to the possibility of further recovery of valuable products. In addition, there is no need to add chemicals [202], the process is selective, and the operating costs are low. With the recovery of the metals, the water is purified by means of the electroactive species formed.

Like any process, reductive electrochemical methods have a disadvantage. The efficiency of the electrochemical process is sensitive to the composition of wastewater and easily interferes with the side reactions of hydrogen generation and oxygen reduction. Wastewater treatment with Hg^2+^, Pb^2+^, Cd^2+^, and Cu^2+^ is usually conducted under acidic conditions at mixed carbon and sulfur cathodes with a different C:S ratio ranging from 20:80 to 80:20. In this case, the metals precipitate in the form of insoluble sulfides or bisulfides, which are later removed from the mechanical system.

To maximize the removal of metal ions from contaminated wastewater, electrical potential was used to modify conventional chemical precipitation into electrochemical precipitation, also known as electrocoagulation [203,204,205,206].

Electrocoagulation is based on the physicochemical process of coagulation of colloidal systems under the action of a continuous electric current [207,208,209]. During the electrolysis of wastewater with steel or aluminium anodes, the electrochemical dissolution of the anode metal takes place [208]. The dissolved aluminium and iron cations are hydrolyzed and act as coagulation agents, which initiate the adhesion and fusion of the particles. In general, coagulation means loss of stability of aggregates in dispersed systems, leading to phase separation. A wide range of pollutants can be removed from water by electrocoagulation, including pathogenic microorganisms, cyanobacteria, and other inorganic colloids [202,210,211,212,213,214].

Electrodialysis is a membrane separation process under the action of an electric field, in which ions are selectively transported through ion exchange membranes [200,215]. The method is selective with high separation efficiency, which makes it suitable for desalination and other treatments with significant environmental benefits [216,217,218,219,220].

Membrane electrolysis is a chemical process driven by an electrolytic potential; it can also be applied to remove metal impurities from wastewater resulting from metal finishing processes. There are two types of cathodes used: a conventional metal cathode (electrowinning) and a large surface cathode [221]. When the electric potential is applied to an ion exchange membrane, the reduction–oxidation reaction takes place in the electrodes [220,222,223,224].

In Table 10, some electrochemical techniques for metal ion removal and the efficiency of the processes are summarized.

*Critical viewpoint for electrochemical treatment technique:*

- For treating large volumes of low-conductivity wastewater, electrochemical techniques are less economically competitive than conventional biological or chemical methods.
- During the process, oxide layers or scale deposits (passivation) form on the surface of the electrodes, which electrically insulate the electrode. This leads to a drastic decrease in efficiency and requires frequent chemical cleaning or polarity reversal.
- Many processes require electrodes made of expensive or special materials (such as titanium coated with metal oxides or doped diamond).
- Even in the case of electrocoagulation, the iron or aluminum anodes wear out quickly and must be replaced periodically, generating maintenance costs.
- Electrochemical processes are often faster but require an initial investment and much higher energy costs, which makes them more suitable for highly concentrated industrial effluents.
- Treatment efficiency depends on pH, salinity, and water composition, which can vary significantly in real industrial effluents, requiring pretreatment or complex, multi-stage treatment systems.

### 2.6. Solvent Extraction Used for Metal Ions Removal from Wastewater

**Figure 11:**
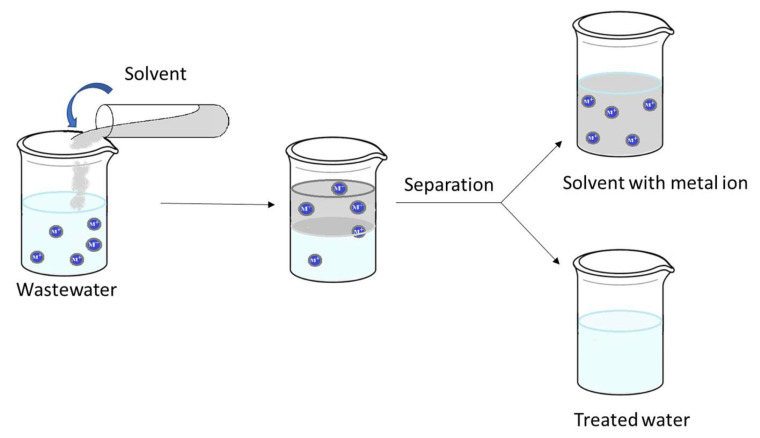
Solvent extraction process scheme.

Solvent extraction (Figure 11) means the transfer of soluble contaminants from wastewater into the solvent. Desirable properties of suitable solvents are: (i) low solubility and miscibility in water (e.g., light crude oil, toluene, pentane and hexane); (ii) greater adsorption capacity of the contaminant than of the water; (iii) easy separation of solvent and wastewater (due to the large density difference); (iv) easy separation of contaminants; (v) low toxicity; (vi) thermal stability.

Solvent extraction is used on a wide range of organic contaminants and metal complexes when a suitable solvent is available and when the concentration of the contaminant is not too low. The method requires simple equipment, so the operation is easy; it has good selectivity and high recovery efficiency of metal ions [230,231]. At low concentrations, solvent extraction is not adequate; adsorption or biological treatment is recommended. Solvent extraction can often be used as a pretreatment step before adsorption or biological treatment [232].

Solvent extraction works by transferring solutes from an aqueous phase to an immiscible organic phase using specific extractants. The separation efficacy depends on the formation of hydrophobic complexes that allow metal ions to transfer from the aqueous phase to the organic phase. Also, by replacing the aqueous phase with polar organic or ionic solvents, researchers are expanding the scope of solvent extraction to bypass traditional solubility limitations and enhance metal ion solvation, such as Nonaqueous Solvent Extraction (NASX) or Hydrophobic Deep Eutectic Solvents (HDESs) that are considered as environmentally friendly alternatives to traditional, toxic organic solvents in extraction and separation processes.

Recent research explores NASX and the use of ionic liquids or deep eutectic solvents (DESs) to enhance selectivity and sustainability [233]. The HDESs (i.e., menthol, thymol, fatty acids, and quaternary ammonium salt) are extensively used for extracting lipophilic compounds, such as volatile fatty acids, bioactive compounds, and metal ions from aqueous phases, as well as wastewater treatment.

In Table 11, some solvent performance in solvent extraction techniques for wastewater treatment are summarized.

*Critical viewpoint for solvent extraction technique:*

- Although effective, many solvents are derived from petroleum and are toxic, flammable, and volatile, posing serious risks to human health and local ecosystems.
- The synthesis of environmentally friendly solvents (i.e., ionic liquids or eutectic solvents) is often hampered by the complexity of the synthesis itself or the high cost.
- Complex wastewater streams containing multiple metal ions can significantly reduce the selectivity of the extractant, making it difficult to purify a single target metal without a pretreatment step.
- Formation of a third phase: this is an undesirable phenomenon in which the organic phase splits into two components: a light phase, rich in diluent, and a viscous “heavy” phase (third phase), rich in extractant and metal complex. It can cause massive plant downtime.
- It is difficult to automate due to the fluid dynamics of the two-phase mixing.
- It requires constant high-energy agitation to maintain extraction efficiency.

### 2.7. Biological Methods Used for Metallic Ion Removal from Wastewater

**Figure 12:**
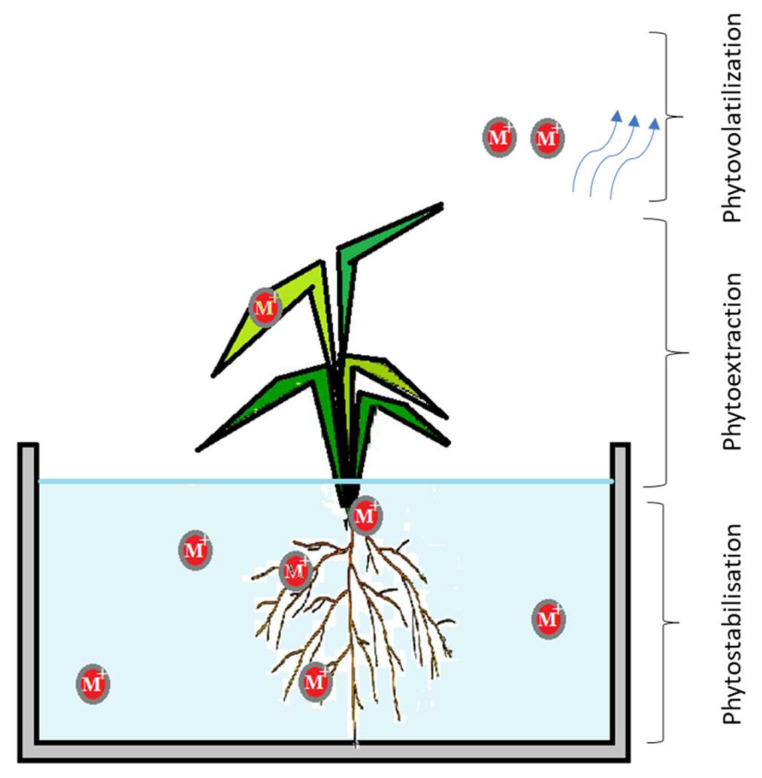
Bioaccumulation process.

Biological agents such as plants or microorganisms offer easy and environmentally friendly ways to remove metal species from wastewater; therefore, they are considered effective and alternative tools. The “core” of the biological method includes phytoextraction (using living plants), bioaccumulation (living microorganisms), or biomineralization (metabolically induced precipitation), which requires living systems to take up, transform, or sequester metals actively. These methods are key, active, and eco-friendly bioremediation techniques that leverage the metabolic processes of living systems to remove heavy metals and pollutants from wastewater. These methods are often preferred over conventional, energy-intensive chemical treatments due to their sustainability. So, bioremediation involves the adsorption, reduction, or removal of contaminants from the aqueous environment through biological resources (Figure 12) that tolerate a high concentration of metal ions [69].

By phytoextraction, plants actively absorb metal cations from water. The metals are translocated through the xylem and sequestered in vacuoles, cell walls, or other non-metabolically active parts of the plant tissues to minimize toxicity. The bioaccumulation process involves living microorganisms (bacteria, fungi, microalgae) that absorb and concentrate heavy metals or organic contaminants inside their cellular structure. This is an active, metabolism-dependent process. Microbes transport metal ions across the cell membrane into the cytoplasm, where they are sequestered by intracellular ligands, such as proteins and peptide ligands. When microorganisms induce the precipitation of metals from wastewater into solid, stable mineral forms, such as carbonates or phosphates, reducing their bioavailability and toxicity, the mineralization occurs.

The remedial properties of metal ions by microorganisms have originated from their self-defence mechanisms, such as enzyme secretion, cell morphological changes, or other mechanisms [239]. In this context, microorganisms play a role in the settlement of solids in solutions, but the process is more a biological adsorption than a true bioremediation technique because the metals are sequestered rather than chemically transformed or removed from the system. Activated sludge, trickling filters, and stabilization ponds are widely used for wastewater treatment. Activated sludge is the most common option that uses microorganisms in the treatment process to break down the organic material through aeration and agitation, which allows the solids to settle. It is continuously recirculated in the aeration basin to increase the rate of organic decomposition [54].

Also, biosorption is a sustainable and cost-effective alternative to remove metal ions particularly from dilute industrial effluents and wastewater. It utilizes biological materials (bacteria, fungi, algae, or agricultural waste) to passively retain metals through mechanisms like ion exchange, surface complexation, and precipitation [240]. Recently, research for the removal of metal ions from industrial effluents by biological methods has focused on the use of agricultural by-products as adsorbents [241]. In this condition, new resources of vegetable waste, such as hazelnut shell, rice husk, walnut shell, jack fruit, corn cob, and rice straw, can be used after chemical modification or conversion by heating into activated carbon or biochar, as adsorbent materials for the adsorption of metal ions from wastewater [240,242]. Biopolymers have functional groups, such as hydroxyls and amines, that increase the efficiency of metal ion adsorption [243]. They are widely used in industry. New materials based on some polysaccharides as derivatives of chitin, chitosan, and starch are described as biopolymers with adsorbent properties for the removal of metal ions from wastewater. But the adsorption mechanisms are complicated and depend on pH [243]. In Table 12, some biological species used in metal ion removal are summarized.

*Critical viewpoint for biological methods:*

- Although extremely efficient on a laboratory scale, limited progress has been made in implementing these methods on a large industrial scale.
- The biggest drawback is the need for a very large volume reactor (bioreactor), as biological methods involve slow metal ion absorption processes compared to advanced chemical methods.
- Many promising studies are limited to synthetic solutions with a single metal, as complex industrial effluents are difficult to manage.
- The efficiency of biological methods depends largely on parameters such as pH, temperature, and nutrient availability, which are extremely difficult to maintain constant in industrial environments.
- Often high concentrations of metals can be toxic to microorganisms themselves, disrupting their metabolic processes and reducing the efficiency of the treatment. Homeostasis is essential in the case of metal ions such as Cu, Zn, and Fe, which are part of enzymatic cofactors or have metabolic roles.

## 3. Evaluation of Metal Ions Removal by Different Physico–Chemical and Biological Treatments

The removal of metal ions from liquid effluents, especially industrial ones, is a very important part of the research carried out in the field of environmental protection. Researchers have developed several methods that include physical, chemical, and biological methods for the removal or recovery of metal-ion species.

To evaluate the performance of all the removal or treatment methods described above in Table 13 a useful comparison is presented for the general evaluation of the removal performance of each method.

## 4. Conclusions

If, at the beginning, the field of wastewater treatment was considered only from a technological point of view (only for chemists, biotechnologists, microbiologists, etc.) and its practical evolution was achieved only by finding new treatment technologies, nowadays, this field has become interdisciplinary. The issue of wastewater treatment is extremely important for the development of human communities, ecosystems, and the preservation of the environment.

As a result of increased awareness in recent years of the importance of protecting and improving water quality, there has been considerable interest in finding more reliable solutions for the removal or recovery of metal ions generated by various industrial processes. Because of this concern, new technologies have been developed and are now available to remove toxic materials from polluted water at reasonable costs for many industrial processes, thus, preventing the pollution of surface water and municipal sewage systems.

Perspectives on the removal of metal ions from wastewater should include combining scientific aspects based on conventional chemical engineering approaches and biotechnological principles with the use of innovative techniques based on ecological principles. The focus must be on cost-effectiveness, environmental sustainability, and high-efficiency disposal using the instrument of artificial intelligence (AI) in the concept of the methods. In this regard, future technologies in water treatment could enable the use of AI together with the Internet of Things (IoT) to create intelligent water quality monitoring systems, thereby increasing operational efficiency and enabling the design of new materials with targeted properties. Advanced AI tools will enable real-time evaluation of treatment process performance, ensuring superior traceability of treatment methods. Similarly, AI should predict phenomena such as membrane fouling or adsorbent capacity depletion, allowing preventive interventions that reduce operating costs. AI algorithms should analyze complex characterization data (such as morphology, specific surface area, or surface chemistry) to understand and control the manufacturing processes of nanocomposite materials used in water treatment and estimate their adsorption performance. Through AI-assisted computational simulations, researchers should identify optimal combinations of functional groups and porous structures to increase selectivity towards specific metal ions. AI should permit automatically adjusted operational parameters (such as pH, adsorbent dosage, time, temperature, or electric current density) depending on fluctuations in the wastewater stream or energy consumption, addressing one of the main criticisms of these technologies. A major gap identified in current research is the low performance of methods on real wastewater compared to synthetic ones. Data from real industrial effluents should be processed through AI to predict how chemical interferences affect removal mechanisms. Future developments in AI should facilitate the transition from small-scale experiments to pilot-scale processes, assessing sustainability and economic feasibility before large-scale implementation.

## Figures and Tables

**Table 1:** The most common heavy metals—use, sources, limits allowed in the aqueous environment (WHO Recommendation).

Metal Ion (M^n+^)	Use	Toxic Effect	Anthropogenic Sources	Natural Sources	WHO Limit (µg/L) [28]
Cadmium (Cd^2+^)	Batteries made of nickel-cadmium, parts for nuclear reactors, laptops, computers, mobile phones, and televisions	Bronchial and pulmonary adenocarcinomas [29], cancers (lung, breast, kidney, prostate, pancreas, nasopharynx) [30], increased bone resorption and a low bone mineral density in children [31] renal and lung disturbances [32]	Iron and steel production, incineration, waste batteries, wastewater treatment sludge from the paint industry, and zinc smelting	Greenockite (cadmium sulfide) mineral, Coal burning, volcanic activities [33]	3
Mercury (Hg^+^, Hg^2+^)	Batteries, fluorescent lights, thermometers, barometers, felt manufacturing, catalysts, and color paints [34]	Illnesses of the nervous system, heart, immune system, genes, development, metabolism, and endocrinology [26,32,35,36]	Thermal power plants, used batteries [34]	Geological deposits of mercury (cinnabar ore), volcanoes, its volatilization from the oceans [34]	6
Lead (Pb^2+^)	Batteries for automobiles, paint and ink pigments, ammunition, diving belts, lead crystal glass, and stained glass for architecture	Anemia, cardiovascular and renal disorders, neurological, cognitive, and biological disorders, endocrine and neuropsychiatric disorders [21,29,32,37] mental and physical disorders in children [29]	Batteries, coal-fired power plants, paints, smelting operations [37]	Mineral—galena, volcanic eruptions, forest fires [37]	10
Arsenic (As^3+^, As^5+^)	Rat poisoning, electronics in the glass and woodworking industries	High blood pressure, rhythm abnormalities, atherosclerosis and consequent atherothrombotic events [21], fatty liver disease, cirrhosis and fibrosis, non-cirrhotic intrahepatic portal hypertension, hepatitis, and neoplastic proliferations of the bladder, kidney, lung, liver, or skin [38,39]	Smelting operations, thermal energy, fuel combustion [26]	Earth’s crust, realgar ore, arsenopyrite, deep sea, geothermal processes, volcanoes [39]	10
Nickel (Ni^2+^)	Manufacture of batteries, catalysts, pigments, alloys, catalyst for hydrogenating vegetable oils [40]	Carcinogenic (lung, nasal cancer), lung fibrosis, allergies, [21,41]	Smelters, power and thermal plants, non-ferrous and ferrous metal processing, galvanization, and the nickel-cadmium battery industry [41,42]	Pentlandite minerals (iron/nickel sulfides), forest fires, volcanic eruptions, wind [40]	70
Chromium (Cr^3+^, Cr^6+^)	Glass pigments, textile production, leather tanning, galvanizing, stainless steel, and different alloys	Chronic exposure to chromium change the cellular epigenetic profile, Cr(VI) being carcinogenic [21] burns and sores (particularly in the stomach and small intestine), anemia, skin rash, allergic reactions, and problems with the reproductive system [43,44]	Combustion gases, mine waters, chromium salt production, tannin manufacturing for the leather sector, and dye production [44,45]	Soils, volcanic, especially like chromite ore (FeCr_2_O_4_) [46]	50 (for total chromium)
Copper (Cu^2+^)	Electrical wiring, furnaces, copper alloys and coins, algicide for water purification, agricultural poison, inks blue pigment	Stomach discomfort, learning and memory problems, and irritations of the nasal, oral, and ocular mucosa [47]	Mining, galvanizing, metallurgy [48]	Different ores: chalcopyrite and bornite vegetable anaerobic decomposition, woods fire	2000
Zinc (Zn^2+^)	Alloys (brass), solders, wood protection, paints batteries [49]	Interact with enzymes and nucleic acids; when it occurs in excess, it can lead to respiratory disorders, acute gastrointestinal disorders, epigastric pain, and disruptions in the balance of many biological systems [50]	Metallurgy, fertilizers, galvanic coatings	Minerals: blende (zinc sulfide) and calamine (zinc silicate), volcanic ash, forest fires, and dust [51]	3000

**Table 2:** Metal ion removal using chemical precipitation and coagulation–flocculation.

Metal Ion (M^+^)	Precipitating Agent	Removal Method	Removal Conditions	Efficiency (%)	References
Cu^2+^	Ca(OH)_2_	Chemical precipitation	pH = 8.73 Ci = 18 mg/L	99.9	[60]
	NaOH	Chemical precipitation	pH = 9.15 Ci = 18 mg/L	99.9	
	Na_2_CO_3_	Chemical precipitation	pH = 8.85 Ci = 18 mg/L	99.9	
Zn^2+^	Ca(OH)_2_	Chemical precipitation	pH = 8.73 Ci = 10 mg/L	99.1	
	NaOH	Chemical precipitation	pH = 9.15 Ci = 10 mg/L	99.7	
	Na_2_CO_3_	Chemical precipitation	pH = 8.85 Ci = 10 mg/L	99.2	
Cu^2+^	Ca(OH)_2_	Chemical precipitation	pH = 11.3 Ci = 100 mg/L	99.9	[62]
	Na_2_S	Chemical precipitation	pH = 10.4 Ci = 100 mg/L	99.8	
	Na_2_CO_3_	Chemical precipitation	pH = 9.3 Ci = 100 mg/L	99.8	
Pb^2+^	Ca(OH)_2_	Chemical precipitation	pH = 6–8 Ci = 64 mg/L	76.1	
	Na_2_S	Chemical precipitation	pH = 6–8 Ci = 64 mg/L	99.7	
	Na_2_CO_3_	Chemical precipitation	pH = 6–8 Ci = 64 mg/L	97.7	
Zn^2+^	Ca(OH)_2_	Chemical precipitation	pH = 10.4 Ci = 100 mg/L	99.6	
	Na_2_S	Chemical precipitation	pH = 9.3 Ci = 100 mg/L	99.8	
	Na_2_CO_3_	Chemical precipitation	pH = 10.4 Ci = 100 mg/L	99.9	
As^5+^	Polyaluminum chloride	Chemical coagulation	pH = 8 Ci = 423 mg/L	99.8	[70]
As^5+^	FeSO_4_∙7H_2_O	Chemical coagulation—precipitation	pH = 9 Ci = 100 mg/L	93.2	[71]
	FeCl_3_∙6H_2_O		pH = 9 Ci = 100 mg/L	75.0	
	Al_2_(SO_4_)_3_∙18H_2_O		pH = 9 Ci = 100 mg/L	37	
Cu^2+^	Poly-ferric sulfates	Coagulation-flocculation	pH = 10–11.5 Ci = 20 mg/L	99.6	[72]
Cu^2+^	Polyacrylamide	Coagulation-flocculation	pH = 10–11.5 Ci = 20 mg/L	95.0	[73]

**Table 3:** Metal ions removal using different flotation agents.

Metal Ion (M^+^)	Flotation Agent	Removal Conditions	Efficiency (%)	References
Cu^2+^	Fe(OH)_3_	pH = 7.5 Ci = 4 mg/L	98.3	[84]
Ni^2+^		pH = 7.5 Ci = 4 mg/L	98.6	
Zn^2+^		pH = 7.5 Ci = 4 mg/L	98.6	
Pb^2+^	Sodium alginate	pH = 5.35 Ci = 83 mg/L	99.0	[85]
Cu^2+^		pH = 8 Ci = 5 mg/L	92.0	
As^5+^	Octanoyl-cysteine	pH = 8 Ci = 5 mg/L	99.5	[86]
Hg^2+^		pH = 8 Ci = 5 mg/L	99.9	
Pb^2+^		pH = 8 Ci = 5 mg/L	99.4	
Cd^2+^		pH = 8 Ci = 5 mg/L	99.2	
Cr^6+^		pH = 8 Ci = 10 mg/L	99.7	
Cu^2+^	Sodium dodecyl sulfate (SDS)	pH = 10 Ci = 10 mg/L	74.0	[87]
Cu^2+^	Hexadecyltrimethyl ammonium bromide (HTAB)	pH = 10 Ci = 10 mg/L	90.0	
Pb^2+^	Bio-surfactants-colloidal gas	pH = 10 Ci = 100 mg/L	90.7	[88]
Cu^2+^		pH = 10 Ci = 30 mg/L	88.2	
Zn^2+^	Sodium dodecyl sulfate (SDS)	pH = 4.5 Ci = 50 mg/L	99.0	[89]
Cu^2+^		pH = 4.5 Ci = 50 mg/L	97.0	
Cr^6+^		pH = 4.5 Ci = 50 mg/L	80.0	
Cu^2+^	Sodium N-lauroylsarcosinate (LS)	pH = 4.5 Ci = 25.6 mg/L	96.3	[90]
Pb^2+^		pH = 4.5 Ci = 82.8 mg/L	99.8	
Cr^3+^		pH = 4.5 Ci = 20.8 mg/L	94.4	
Cd^2+^	Sodium dodecyl sulfate (SDS)	pH = 8 Ci = 63 mg/L	98.2	[91]
Pb^2+^		pH = 8 Ci = 134 mg/L	99.9	
Cu^2+^		pH = 8 Ci = 46 mg/L	97.5	
Cr^3+^		pH = 8 Ci = 87 mg/L	96.9	
Ni^2+^		pH = 8 Ci = 183 mg/L	94.4	

**Table 4:** Summary of the highest reported adsorption capacities by carbon-based adsorbents for metal ion removal.

Metal Ion (M^n+^)	Carbon-Based Adsorbents	Removal Conditions	Efficiency (%)	References
Biochar				
Pb^2+^	Biochar in acidic solution	pH = 5.5–6 Ci = 200 mg/L	88.7	[110]
Cd^2+^		pH = 5.5–6 Ci = 200 mg/L	96.8	
Zn^2+^		pH = 5.5–6 Ci = 200 mg/L	96.2	
Cu^2+^		pH = 5.5–6 Ci = 200 mg/L	93.3	
Co^2+^		pH = 5.5–6 Ci = 200 mg/L	95.9	
Activated carbon				
Pb^2+^	Commercial activated carbon	pH = 5.5–6 Ci = 200 mg/L	54.6	[110]
Cd^2+^		pH = 5.5 Ci = 200 mg/L	68.0	
Zn^2+^		pH = 5.5 Ci = 200 mg/L	63.0	
Cu^2+^		pH = 5.5 Ci = 200 mg/L	80.5	
Co^2+^		pH = 5.5 Ci = 200 mg/L	36.7	
Cu^2+^	ZnCl_2_-activated carbon	pH = 6 Ci = 43.5 mg/L	78.1	[116]
Fe^2+^		pH = 6 Ci = 16.6 mg/L	84.1	
Zn^2+^		pH = 6 Ci = 17.4 mg/L	34.7	
Pb^2+^		pH = 6 Ci = 0.005 mg/L	100	
Charcoal				
As^5+^	Unmodified charcoal	pH = 8.3 Ci = 5 mg/L	42.0	[117]
As^3+^		pH = 8.3 Ci = 5 mg/L	62.5	
Cr^6+^		pH = 8.3 Ci = 50 mg/L	19.6	
Hg^2+^		pH = 8.3 Ci = 5 mg/L	97.0	
As^5+^	FeCl_3_-modified charcoal	pH = 8.3 Ci = 5 mg/L	99.9	
As^3+^		pH = 8.3 Ci = 5 mg/L	99.9	
Cr^6+^		pH = 8.3 Ci = 50 mg/L	99.9	
Hg^2+^		pH = 8.3 Ci = 5 mg/L	99.9	

**Table 5:** Summary of the highest reported adsorption capacities of materials with inorganic support structures.

Metal Ion (M^n+^)	Adsorbent	Removal Conditions	Adsorption Capacity, (mg M^n+^/g Adsorbent)	References
Cs^+^	MgSiO_3_-Cyphos IL-101	pH = 8 Ci = 50 mg/L	3.08	[133]
Cs^+^	SiO_2_-Cyphos IL-101	pH = 8 Ci = 50 mg/L	1.48	[134]
Cs^+^	MgSiO_3_-tioureea	pH = 8 Ci = 50 mg/L	2.10	[135]
Cu^2+^	Silica MCM-41	pH = 5 Ci = 10 mg/L	9.70	[136]
Pb^2+^		pH = 5 Ci = 10 mg/L	18.8	
La^3+^	MgSiO_3_-TBAH2P	pH = 6 Ci = 200 mg/L	9.05	[137]
Pd^2+^	MgSiO_3_-LCys	pH = 3 Ci = 40 mg/L	9.23	[138]
Pd^2+^	MgSiO_3_-DB30C10	pH = 3 Ci = 200 mg/L	21.6	[129]
Pt^4+^		pH = 3 Ci = 140 mg/L	30.0	[139]

**Table 6:** Summary of the highest reported adsorption capacities of materials with polymeric support structures.

Metal Ion (M^n+^)	Adsorbent	Removal Conditions	Adsorption Capacity, (mg M^n+^/g Adsorbent)	References
As^5+^	XAD-7-DEHPA-Fe dry method impregnated	pH = 6–8 Ci = 100 mg/L	0.176	[147]
As^5+^	XAD-8-DEHPA-Fe	pH = 6–8 Ci = 100 mg/L	0.0226	[148]
Zn^2+^	XAD-7-DEHPA	pH = 1–8 Ci = 40 mg/L	5.00	[149]
Cd^2+^		pH = 1–8 Ci = 40 mg/L	4.50	
Cs^+^	Amberjet UP1400	pH = 8 Ci = 100 mg/L	6.36	[150]
	Amberlite IR120	pH = 8 Ci = 100 mg/L	8.67	
La^3+^	XAD7-Na-β-gli-P	pH = 6 Ci = 300 mg/L	31.7	[151]
Nd^3+^		pH = 6 Ci = 300 mg/L	64.7	
Eu^3+^	XAD7-TBAH2P	pH = 6 Ci = 50 mg/L	74.2	[152]
Pt^4+^	XAD7-DB30C10	pH = 6 Ci = 50 mg/L	12.3	[139]
Pt^4+^	XAD7-DB18C6	pH = 3 Ci = 175 mg/L	6.73	[153]
Pd^2+^		pH = 3 Ci = 175 mg/L	6.40	
Ru^3+^		pH = 3 Ci = 175 mg/L	10.7	
Au^3+^	XAD7-AcLG	pH = 3 Ci = 100 mg/L	14.2	[154]
Pb^2+^	XAD-16-dipicolylamine	pH = 5.6 Ci = 1500 mg/L	167	[155]
Cu^2+^		pH = 5.6 Ci = 300 mg/L	36.6	
Cu^2+^	Biochar derived from brinjal stem	pH = 5.6 Ci = 30 mg/L	246.31	[156]
Pb^2+^		pH = 5.6 Ci = 30 mg/L	183.15	
Cr^6+^		pH = 5.6 Ci = 30 mg/L	71.89	
Cd^2+^	Biochar derived from citrus peel	pH = 5.6 Ci = 30 mg/L	15.46	

**Table 7:** Summary of the highest reported adsorption capacities of hybrid composite materials.

Metal Ion (M^n+^)	Adsorbent	Removal Conditions	Adsorption Capacity, (mg M^n+^/g Adsorbent)	References
Cr^6+^	Composite of tungsten trioxide (WO_3_) with polyaniline (PANI)	pH = 2 Ci = 100 mg/L	549.37	[161]
Pb^2+^	Silica conjugate adsorbent (CJA)	pH = 5.5 Ci = 10 mg/L	175.16	[162]
Pb^2+^ single component solution	Tetraethylenepentamine (TEPA) modified chitosan/CoFe_2_O_4_	pH = 5 Ci = 800 mg/L	228.31	[163]
Cu^2+^ single component solution		pH = 5 Ci = 1600 mg/L	168.06	
Cr^6+^	Polyethylenimine modified biochar	pH = 2–7 Ci = 100 mg/L	435.7	[164]
Cu^2+^	Silica—4-tert-Octyl-4-((phenyl)diazenyl)phenol (TPDP)	pH = 4 Ci = 5 mg/L	184.73	[165]

**Table 8:** Performance of some membranes used for metal ion removal.

Metal Ion (M^n+^)	Filtration System Types (Operation Condition)	Removal Conditions	Efficiency (%)	References
Cu^2+^	UF—hybrid matrix membranes	pH = 2 Ci = 10,000 mg/L	92	[76]
Hg^2+^	Poly(vinylidene fluoride) (PVDF)	pH = 1.5 Ci = 400 mg/L	97	[180]
Cu^2+^	MF—graphitic carbon nitride membrane	pH = 5.6 Ci = 2500 mg/L	98	[183]
As^5+^	NF	pH = 8 Ci = 100 mg/L	99.8	[184]
Pb^2+^	UF, different types of membrane	pH = 5–6 Ci = 200 mg/L	90–99	[185]
Pb^2+^	NF, different type of membrane	pH = 5–6 Ci = 200 mg/L	80–99	[186]
Pb^2+^	RO	pH = 4–7 Ci = 500 mg/L	98.54	[187]
Cd^2^		pH = 4–7 Ci = 500 mg/L	97.97	
Pb^2+^	RO	pH = 4–7 Ci = 500 mg/L	99.75	[188]

**Table 9:** Performance of ion exchange resin used for heavy metal removal.

Metal Ion (M^+^)	Ion Exchange Resin Types	Removal Conditions	Efficiency (%)	References
Cu^2+^	Natural zeolites (Clinoptilolites)	pH = 6 Ci = 100 mg/L	90	[191]
	Synthetic zeolites	pH = 6 Ci = 200 mg/L	100	
Cd^2+^	Natural zeolites (Clinoptilolites)	pH = 6.9 Ci = 100 mg/L	90	
	Synthetic zeolites	pH = 6.6 Ci = 200 mg/L	100	
Zn^2+^	Natural zeolites (Clinoptilolites)	pH = 6.7 Ci = 100 mg/L	90	
	Synthetic zeolites	pH = 6.8 Ci = 200 mg/L	100	
Cr^6+^	Amberlite INR 77	pH = 2–8 Ci = 100 mg/L	98	[192]
	SKN1 resin	pH = 2–8 Ci = 100 mg/L	98	
Cr^3+^	Amberjet 1200H	pH = 2–6 Ci = 10 mg/L	100	[193]
	Amberjet 1500H	pH = 2–6 Ci = 10 mg/L	100	
	Amberjet IRN97H	pH = 2–6 Ci = 10 mg/L	100	
Cr^3+^	ZGC351 resin coated with chitosan	pH = 2–6 Ci = 480 mg/L	31	[194]
Cd^2+^		pH = 2–6 Ci = 48 mg/L	52	
Pb^2+^		pH = 2–6 Ci = 1000 mg/L	31	
Sr^2+^		pH = 2–6 Ci = 27 mg/L	52	
Pb^2+^	Amberlite IR 120	pH = 2–6 Ci = 100 mg/L	99	[195]
Zn^2+^	Lewatit OC-1026	pH = 1–4 Ci = 100 mg/L	99	[196]
Ni^2+^	Dowex 50WX8	pH = 4–6 Ci = 100 mg/L	83.3	[197]
Cd^2+^		pH = 4–6 Ci = 100 mg/L	95.1	
Pb^2+^		pH = 4–6 Ci = 100 mg/L	96	
Zn^2+^		pH = 4–6 Ci = 100 mg/L	100	
Cu^2+^		pH = 4–6 Ci = 100 mg/L	82.3	

**Table 10:** Performance of the electrochemical process used for metal ion removal.

Metal Ion (M^n+^)	Electrochemical Process	Removal Conditions	Efficiency (%)	References
Cr^6+^	Electrochemical reduction	pH = 4–6 Ci = 500 mg/L	100	[225]
Pb^2+^	Electrodialysis	pH = 5 Ci = 10 mg/L	99.9	[226]
Ni^2+^		pH = 5 Ci = 10 mg/L	96.9	
Cr^6+^	Electrodialysis	pH = 2.2–8.5 Ci = 100 mg/L	99.0	[227]
Cr^3+^	Electrocoagulation	pH = 9.5 Ci = 100 mg/L	100	[228]
Cu^2+^		pH = 9.5 Ci = 30 mg/L	99.0	
Ni^2+^		pH = 9.5 Ci = 60 mg/L	98.0	
Zn^2+^		pH = 9.5 Ci = 20 mg/L	99.0	
Cu^2+^	Electrodeionization	pH = 4–6 Ci = 500 mg/L	93.2	[229]
Cr^6+^		pH = 4–6 Ci = 50 mg/L	99.0	
Cr^3+^		pH = 4–6 Ci = 50 mg/L	99.0	
Cu^2+^	Bioelectrochemical	pH = 4–6 Ci = 1000 mg/L	99.9	
Cr^3+^	Photoelectrochemical	pH = 4–6 Ci = 10 mg/L	100	
Cu^2+^		pH = 4–6 Ci = 12 mg/L	100	
Pb^2+^		pH = 4–6 Ci = 40 mg/L	101	

**Table 11:** Performance of solvent extraction method used for metal ion removal.

Metal Ion (M^n+^)	Solvent Extraction	Removal Conditions	Efficiency (%)	References
Cu^2+^	2-hydroxy-5-nonylacetophenone oxime (Mextral 84H) bis(2,4,4-trimethylpentyl)-phosphinic acid (Cyanex 272)	pH = 4–6 Ci = 10 mg/L	100	[230]
Ni^2+^		pH = 4–6 Ci = 10 mg/L	100	
Zn^2+^		pH = 4–6 Ci = 10 mg/L	98.0	
Cd^2+^		pH = 4–6 Ci = 10 mg/L	98.0	
Ir^4+^	HDES single-stage extraction in oleic acid oscillation time 20 min	pH = 3 Ci = 20 mg/L	97.57	[231]
Ru^3+^		pH = 3 Ci = 20 mg/L	94.26	
Pt^4+^		pH = 3 Ci = 20 mg/L	99.55	
Rh^3+^		pH = 3 Ci = 20 mg/L	15.1	
Cu^2+^		pH = 3 Ci = 20 mg/L	100	
Fe^3+^		pH = 3 Ci = 20 mg/L	99.8	
Zn^2+^	10% Aliquat 336	pH = 1.45 Ci = 10 mg/L	99.9	[232]
Cu^2+^	5 vol.% LIX 984N-C	pH = 1.2 Ci = 10 mg/L	100	
Fe^3+^	10% DEHPA	pH = 5.25 Ci = 10 mg/L	100	
Cr^3+^	Alamine 336-xylene (Simultaneous Extraction)	pH = 4–5 Ci = 60 mg/L	90.6	[234]
Cd^2+^		pH = 4–5 Ci = 8 mg/L	99.4	
Zn^2+^		pH = 4–5 Ci = 14 mg/L	98.7	
Cr^3+^	Alamine 336-xylene (Selective Extraction)	pH = 4–5 Ci = 60 mg/L	89.8	
Cd^2+^		pH = 4–5 Ci = 8 mg/L	98.9	
Zn^2+^		pH = 4–5 Ci = 14 mg/L	89.0	
Cr^6+^	Amides	pH = 4–5 Ci = 50 mg/L	95.4	[235]
Pb^2+^	HDES (thymol and decanoic acid)	pH = 4–5 Ci = 50 mg/L	98.91	[236]
Cu^2+^	HDES (dodecanoic acid and octanoic acid molar ratio 1:3)	pH = 5–7 Ci = 20 mg/L	85.61	[237]
Co^2+^		pH = 5–7 Ci = 20 mg/L	96.19	
Ni^2+^		pH = 5–7 Ci = 20 mg/L	76.54	
Fe^3+^	HDES (Aliquat 336:L-menthol, molar ratio 3:7)	pH = 3–5 Ci = 560 mg/L	99	[238]
Mn^2+^		pH = 3–5 Ci = 550 mg/L	99	
Co^2+^		pH = 3–5 Ci = 600 mg/L	99	

**Table 12:** Performance of some biological methods used for metal ion removal.

Metal Ion (M^n+^)	Biological Species	Removal Conditions	Bioaccumulation (mg/kg d.w.)	References
Zn^2+^	*Typha latifolia* After 45 days of treatment	pH = 7 Ci = 44 mg/L	271	[244]
Cu^2+^		pH = 7 Ci = 8.63 mg/L	47.0	
Zn^2+^	*Thelypteris palustris* After 45 days of treatment	pH = 7 Ci = 44/L	409	
Cu^2+^		pH = 7 Ci = 8.63 mg/L	105	
Cu^2+^	*Thevetia peruviana*	pH = 5 Ci = 50 mg/L	187.51	[245]
Pt^2+^	*Escherichia coli*	pH = 2–5 Ci = 450 mg/L	239.92	[246]
Cr^6+^	*Quercus crassipes* corn shell	pH = 1–2 Ci = 400 mg/L	110.35	[247]
Pb^2+^	*Ludwigia stolonifera*	pH = 2–10 Ci = 100 mg/L	2440	[248]
Cr^6+^		pH = 2–10 Ci = 100 mg/L	7001	

**Table 13:** Comparative evaluation of the performance of methods for removing metal ions from water.

Treatment Processes	Advantage	Disadvantage
Precipitation, coagulation-flocculation, sedimentation or flotation processes	Removal of heavy metals from wastewater, preferably for higher concentrations, Coagulation-flocculation—less sludge settling, dewatering	Avoiding agents that can lead to the formation of complexes, pH adjustment is important, It cannot be applied at concentrations lower than the solubility of the precipitate, Requires sludge storage, Odor emissions are possible, Coagulation filtration -costly and high consumption of chemicals
Adsorption processes	Recovery is possible, Low adjacent space requirements, The process can be automated, GAC High Efficiency Elimination of non-biodegradable or toxic substances	Applicable for low pollutant concentrations, or when guaranteed recovery is desired, The adsorbent used must be regenerated (high energy consumption) or stored (causing the need to incinerate the waste), GAC Performance depends on adsorbent, costly, no regeneration.
Filtration membranes	Through reverse osmosis a high degree of purity is achieved to recycle and reuse water and high concentrations of contaminants that require further treatment is generated, MF—high separation selectivity, low space requirement, NF—high separation efficiency, easy operation, reliability.	Concentrations with too high an osmotic pressure to function, Low thermal and chemical resistance, RO membranes are very sensitive to fouling and under harsh operating conditions, it can lead to membrane degradation and physical damage. It requires a reliable pre-treatment system to guarantee acceptable performance and high durability of membrane in RO MF—maintenance and operation cost high, NF—when compared to UF, it has a low anti-compacting ability.
Ion exchange processes	Regeneration of materials, selective for metal ions.	Accessible for a smaller number of metal ions, expensive.
Electrochemical processes	Selective for metal ions, no chemical consumptions, most of the metal ions possible to remove	High current density requirement, high operational and capital cost.
Solvent extraction processes	Activates the removal of refractory or toxic organic compounds and some metals.	Residues must be stored or incinerated, Limited application due to solvent characteristics.
Biological processes	High concentrations can be reduced due to high conversion of microorganisms, Suitable for high concentrations of compounds containing sulfur, chlorides or nitrogen.	Biomass structures must be removed because as waste they can cause blockage in the aqueous environment, Hardly soluble components are more difficult to reduce, The excessive fluctuation of pollutant concentrations has a great influence on the performance of the process, Leaching water must be treated separately.

## Data Availability

No new data were created or analyzed in this study.
